# Sea Vegetables and Fruits as Novel Dietary Protective Factors for Sarcopenia and Muscle Function in Taiwan: A Cross-Sectional Study

**DOI:** 10.3390/nu17233805

**Published:** 2025-12-04

**Authors:** Chi-Hsien Huang, Pei-Fang Li, Tzyh-Chyuan Hour, Huei-Mei Chen, Hsin-Yi Chang, Yu-Kuei Chen

**Affiliations:** 1 Department of Family Medicine and Community Medicine, E-Da Hospital, I-Shou University, Kaohsiung 82445, Taiwan; ed103520@edah.org.tw; 2 College of Medicine, I-Shou University, Kaohsiung 82445, Taiwan; 3 College of Nursing, Kaohsiung Medical University, Kaohsiung 80708, Taiwan; 4 Nutrition Division, Pingtung Hospital, Ministry of Health and Welfare, Pingtung 90003, Taiwan; pa8018pa@yahoo.com.tw; 5 Department of Biochemistry, School of Medicine, Kaohsiung Medical University, Kaohsiung 80708, Taiwan; cliff@cc.kmu.edu.tw; 6 Department of Medical Research, Kaohsiung Medical University Hospital, Kaohsiung 80708, Taiwan; 7 Department of Nutrition, Pingtung Veterans General Hospital Longquan Branch, Pingtung 912012, Taiwan; lmy2052002@gmail.com; 8 Department of Nutrition, Kaohsiung Veterans General Hospital, Kaohsiung 81341, Taiwan; hychang0923@vghks.gov.tw; 9 Department of Nutrition, I-Shou University, Kaohsiung 82445, Taiwan

**Keywords:** sarcopenia, older Taiwanese adults, sea vegetables, fresh fruits

## Abstract

**Background/Objectives:** Sarcopenia may be influenced by lifestyle and dietary factors. Emerging evidence suggests that certain foods such as sea vegetables and fruits contain bioactive compounds may help protect against muscle loss. This study investigated the association between sea vegetable and fruit intake and the risk of sarcopenia and physical performance in older adults in Taiwan. **Methods:** We conducted a cross-sectional study of 588 individuals aged ≥65 years recruited from three hospitals (outpatient and home-care settings) in southern Taiwan (2018–2020). Questionnaire, medical chart, and laboratory data were used to examine the associations between demographic characteristics, dietary intake, and nutritional status and sarcopenia, defined as low muscle mass plus reduced strength or poor physical performance. Sarcopenia was diagnosed using Asian Working Group for Sarcopenia 2019 criteria. The performance variables we measured were grip strength, gait speed, and chair stand time. Logistic regression was used to identify associated factors, and linear regression was used to assess the contributions of these factors to performance measures. **Results:** Sarcopenia was identified in 159 (27.0%) of the 588 participants. Those with sarcopenia had lower education levels, poorer nutritional status, weaker grip strength, and slower mobility. Daily intakes of sea vegetables (adjusted OR = 0.38, 95% CI: 0.20–0.74) and fresh fruits (adjusted OR = 0.28, 95% CI: 0.16–0.49) were independently associated with reduced risk of sarcopenia. Sea vegetable intake was positively associated with grip strength, while fruit intake was inversely associated with chair stand time. **Conclusions:** Dietary factors and nutritional status were significantly associated with sarcopenia risk and physical performance. Sarcopenia prevention strategies might want to include promoting the consumption of sea vegetables and fruits.

## 1. Introduction

Sarcopenia, defined as an age-related syndrome characterized by a progressive decline in skeletal muscle strength, muscle mass, and physical performance, affects approximately 10–16% of older adults worldwide, with a higher prevalence among patient populations, ranging between 18% in those with diabetes and 66% in those with unresectable esophageal cancer [1]. The European Working Group for Sarcopenia in Older People (EWGSOP) established a currently widely used diagnostic framework in 2010 and updated it in 2018, pinpointing low muscle strength as the key indicator of sarcopenia [2].

Sarcopenia shares certain clinical manifestations with other muscle-wasting conditions, cachexia and malnutrition. Cachexia is a multifactorial metabolic syndrome secondary to chronic diseases such as cancer, heart failure, or chronic obstructive pulmonary disease, and is typically accompanied by systemic inflammation and involuntary weight loss. Malnutrition results from inadequate nutrient intake or absorption and may coexist with, but is not synonymous with, sarcopenia. Sarcopenia is primarily characterized as a progressive and generalized loss of skeletal muscle mass and function due to aging. Distinguishing these conditions is clinically important because their underlying mechanisms, diagnostic criteria, and the therapeutic strategies used to treat them differ.

The factors commonly associated with sarcopenia are male sex, BMI, malnutrition, and osteoarthritis. In addition, the prevalence of sarcopenia varies across settings where there may be differences in nutritional status, calf circumference, smoking habit, and physical inactivity as well as differences in the prevalence of heart disease, diabetes, cognitive impairment, and depression [3]. In northern Taiwan, one study of 173 older adults in eight daycare centers for the elderly in Keelung City reported an alarmingly high prevalence of sarcopenia, with 47.4% classified according to the Asian Working Group for Sarcopenia 2019 (AWGS 2019) criteria as having possible sarcopenia and 50.9% as having confirmed sarcopenia [4]. That study identified male sex, BMI, calf circumference, dementia, and malnutrition risk as significant correlates [4].

Emerging evidence suggests that intake of specific food groups such as sea vegetables, such as seaweed and hair vegetables, and fruits may play a protective role against age-related muscle loss. Sea vegetables are rich in marine-derived compounds, including fucoidan, phlorotannins, and fucosterol, which have been found to have anti-inflammatory and myogenic activities in preclinical models [5,6,7,8]. Likewise, many fruits contain polyphenols such as quercetin and kaempferol, which promote muscle cell migration and differentiation [9,10]. These compounds have been shown to modulate key pathways involved in muscle metabolism, including suppression of oxidative stress, attenuation of inflammatory cytokines that drive muscle catabolism, and activation of anabolic signaling such as the PI3K/Akt–mTOR axis in preclinical models.

One large population-based study from Korea found a dietary pattern rich in white rice, fish, and seaweeds to be associated with a significantly lower prevalence of low muscle mass in middle-aged and older adults [11]. Similarly, a Japanese study using Asian Working Group for Sarcopenia (AWGS) 2019 criteria found a nutrient-dense dietary pattern characterized by higher intakes of fish, vegetables, seaweeds, and fruits to be inversely associated with sarcopenia among community-dwelling older adults [12]. These findings highlight the potential role of these traditional East Asian dietary components, particularly sea vegetables, fish, legumes, and fruits, in supporting muscle health. No study in Taiwan, where the population follows a similar diet, has specifically examined the association between the consumption of sea vegetables and fruits or setting and sarcopenia, and no study has evaluated them together along with nutritional status and functional performance in older adults.

Therefore, we performed a cross-sectional study to investigate the associations of dietary patterns, nutritional status, and institutional factors with sarcopenia and functional performance among older adults in southern Taiwan. We hypothesized that higher intake of sea vegetables and fruits would be associated with a lower risk of sarcopenia and better muscle function. Specifically, this study aimed to (1) examine whether intakes of sea vegetables and fruit are associated with the risk of sarcopenia, (2) evaluate the relationship between these dietary components and physical performance measures such as gait speed and grip strength, and (3) determine how nutritional indicators and dietary patterns relate to sarcopenia and functional outcomes in older adults.

To conduct this study, we recruited 588 participants 65 years and older who had no record of terminal illness, intensive care admission, advanced dementia, complete dependence on caretakers, or incomplete data from three healthcare institutions (2 outpatient departments, 1 home-based care center) in southern Taiwan. On the day each patient was enrolled, we collected anthropologic data, and administered questionnaires to collect demographic and lifestyle data, dietary recall data, and nutritional assessment data using the Mini Nutritional Assessment (MNA). On the same day, we administered performance tests assessing hand grip, gait speed, and functional mobility. Later, we collected recent biochemical data from medical records. All participants were screened for sarcopenia based on AWGS 2019 criteria, which, like EWGSOP, prioritize muscle strength, but unlike EWGSOP, provide cut-off points tailored for Asian populations [3].

## 2. Materials and Methods

### 2.1. Sarcopenia Diagnosis and Functional Measurements

Sarcopenia was diagnosed based on the criteria recommended by the Asian Working Group for Sarcopenia (AWGS) 2019 consensus [13]. Participants were first required to meet the low muscle mass criterion, defined as a skeletal muscle mass index (SMI) below 7.0 kg/m^2^ in men or 5.7 kg/m^2^ in women, as assessed by bioelectrical impedance analysis (BIA). In addition, they had to meet at least one of the following conditions: (i) low muscle strength, defined as handgrip strength < 28 kg for men or <18 kg for women; (ii) poor physical performance, defined as gait speed < 1.0 m/s over a 6 m walk; or (iii) impaired chair stand performance, defined as ≥12 s to complete five rises or inability to complete the test. Participants who did not meet these thresholds were classified as non-sarcopenic.

### 2.2. Nutritional Assessments, Dietary Data, and Biochemical Data

This study used structured questionnaires to collect information on participants’ demographic characteristics and health behaviors, which included smoking, drinking, and betel chewing. The questionnaires also required each participant to recall his or her intake of main foods, snacks, and drinks. For analytical clarity and consistency, specific food items were grouped into broader categories. The key categories were the following: sea vegetables (encompassing various edible algae such as seaweed and hair vegetables); fresh fruits (such as grapes, bananas, lychees, and longan); light-colored vegetables (such as radishes and Chinese cabbage); dried small fish with bones (such as kissed larvae and other small, whole-eaten fish); other non-fish seafood (such as shrimp, flower sticks, and hairy crab); fish paste products (such as fish balls and fish soup); and processed meat products (such as gong balls and hot dogs). This questionnaire collected participants’ dietary intake using a semi-quantitative food frequency approach with frequency of consumption measured across five categories, from less than once per month to at least once per day. Nutritional status was assessed using the Mini Nutritional Assessment (MNA), a validated screening tool for older adults. MNA scores ≥ 24 indicated normal nutritional status, while scores of 17–23.5 identified individuals at risk of malnutrition, requiring early nutritional intervention. Scores < 17 indicated protein-energy malnutrition, necessitating a comprehensive nutritional assessment, including serum albumin and prealbumin levels, and dietary intake records.

### 2.3. Data Source, Participants, and Study Design

This cross-sectional study included 588 participants aged 65 years and older recruited between September 2018 and May 2020. Prior to recruitment, we checked the medical records of all patients and excluded those who had severe or terminal illness, intensive care admission, advanced dementia, complete dependence on caretakers for daily activities, or incomplete data. Participants were purposively sampled from three hospitals in southern Taiwan: Kaohsiung Veterans General Hospital (KVGH), Pingtung Veterans General Hospital Longquan Branch, and Pingtung Hospital. At KVGH and Pingtung Hospitals, the participants were recruited from the outpatient clinics of the endocrinology and metabolism departments. At Pingtung Veterans General Hospital Longquan Branch, the participants were recruited from the home care center.

This study was supported by Kaohsiung Veterans General Hospital (Grant number: KSE107-018), I-Shou University (Grant number: ISU-114-IUC-05), and National Science and Technology Council (Grant number: NSTC 114-2320-B-214-001). The funding organizations had no role in study design, data collection, analysis, interpretation, or the writing of the manuscript. The protocol for this study was approved by the institutional review boards of KVGH (VGHKS18-CT8-18) and Antai Tian-Sheng Memorial Hospital (TSMH19-037-B).

Biochemical blood data were retrieved from the hospital’s electronic medical records. The most recent values available at the time of study enrollment were used. Additionally, the participants’ most recent laboratory test results assessed during outpatient visits and hospital admission were used to identify chronic diseases such as diabetes, hypertension, or hyperlipidemia.

We recorded each participant’s anthropometric measurements, including body mass index (BMI), arm circumference, and skinfold thickness. Body composition was evaluated by bioelectrical impedance analysis (BIA), which estimates the percentage of skeletal muscle mass relative to total body weight.

Handgrip strength was measured using a calibrated dynamometer (Tokyo, Japan). During measurement, participants sat with their feet shoulder-width apart and flat on the floor. Measurements were performed three times with their dominant hand at 30 s intervals; the highest value was used for analysis. Gait speed was assessed over a six-meter walk at each participant’s usual pace, recording the time required to complete the distance. Functional mobility was further evaluated using the timed up-and-go test, wherein participants rose from an armless chair, walked three meters, turned, walked back, and sat down again. Based on total time taken, mobility was categorized as normal (<10 s), mild impairment (10–20 s), moderate impairment (20–29 s), or severe impairment (>29 s).

### 2.4. Statistics

We used the chi-square test (χ^2^) and the independent samples *t*-test to compare the differences between categorical and continuous variables among groups. Fisher’s exact test was used where appropriate. Multivariate logistic regression analysis was conducted to analyze the association between risk factors and sarcopenia. Crude models included unadjusted risk estimates, whereas fully adjusted models took into account potential confounders. Backward stepwise regression was employed for model selection. Multiple linear regression analysis was used to examine the impact of various factors muscle strength, and gait speed. All statistical operations were performed using SAS version 9.4 (SAS Institute, Cary, NC, USA). All tests were two-tailed, and significance was set at *p* < 0.05.

## 3. Results

The macronutrient compositions of the sea vegetables and fruits we focused on in this study are summarized in Table 1. Sea vegetables had profiles characterized as low caloric density, minimal fat, and measurable amounts of dietary fiber. The fruits assessed in this study exhibited macronutrient patterns typical of fresh tropical produce, characterized mainly by their carbohydrate contributions.

A total of 588 participants were recruited. One hundred fifty-nine individuals were diagnosed as having sarcopenia (mean age, 81.1 years) and 429 were not (mean age, 81.8 years). Those with sarcopenia were more likely to be male, though not significantly different from those without. They tended to be less educated (below high school level). They had significantly higher intakes of dried small fish with bones (kissed larvae, dried small fish), canned fish, other non-fish seafood (shrimp, flower sticks, hairy crab), processed meat products (gong balls, hot dogs) compared to non-sarcopenic individuals, but significantly lower intakes of fish paste products (fish balls, fish soup), sea vegetables (algae) (sea vegetables, seaweed, hair vegetables), canned and salted frozen vegetables, and fresh fruits (grapes, bananas, lychees, longan). Biochemically, they had significantly higher levels of uric acid and total cholesterol. We also found significant differences in the prevalence of this disease across recruitment sites (Table 2).

Based on our crude logistic regression model, participants who had daily intakes of light-colored vegetables (radish, Chinese cabbage), sea vegetables (algae) (sea vegetables, seaweed, hair vegetables), and fresh fruits (grapes, bananas, lychees, longan) and those who had a glomerular filtration rate of per 10 min/mL/1.73 m^2^ were significantly less likely to have sarcopenia. Those recruited from Kaohsiung Veterans General Hospital (compared to Pingtung Hospital) were significantly more likely to have sarcopenia. After adjustment for age, sex, education level, dietary habits, nutritional status in the multiple logistic regression model, we found a significant association between daily intakes of sea vegetables (algae) (sea vegetables, seaweed, hair vegetables) and fresh fruits (grapes, bananas, lychees, longan) and a reduced risk of sarcopenia. The odds ratio and 95% confidence intervals were 0.38 (95% CI: 0.20 to 0.74, *p* = 0.004) and 0.28 (95% CI: 0.16 to 0.49, *p* < 0.001), respectively (Table 3).

Our multiple logistic regression model adjusted for age, gender, education level, canned fish, dried small fish with bones (kissed larvae, dried small fish), fish paste products (fish balls, fish soup), other non-fish seafood (shrimp, flower sticks, hairy crab), processed meat products (gong balls, hot dogs), livestock and poultry offal (chicken liver, kidney), light-colored vegetables (radish, Chinese cabbage), sea vegetables (algae) (sea vegetables, seaweed, hair vegetables), canned and salted frozen vegetables, fresh fruits (grapes, bananas, lychees, longan), green tea, Mini Nutritional Assessment (MNA), glomerular filtration rate and enrolled institution.

Multivariable linear regression analysis was conducted to examine the relationship between the factors associated with sarcopenia diagnostic criteria and the dependent variables of hand grip, 6 m gait speed, and chair stand test time. Age, sex, intake of sea vegetables (algae) (sea vegetables, seaweed, hair vegetables), nutritional status, and estimated glomerular filtration rate (eGFR) were significantly associated with grip. Sex, light-colored vegetables (radish, Chinese cabbage) daily, canned fish, fresh fruits (grapes, bananas, lychees, longan) daily, nutritional status, and eGFR were significantly associated with chair stand test time. Notably, sea vegetables (algae) (sea vegetables, seaweed, hair vegetables) were significantly associated with greater grip. After adjustment for age, sex, education level, dietary habits, nutritional status, the regression coefficient for sea vegetables (algae) (sea vegetables, seaweed, hair vegetables) was 1.9 (95% CI: 0.7–3.1, *p* = 0.002) for grip and was 0.2 (95% CI 0.1–0.4, *p* = 0.003) for 6 m gait speed. Additionally, in the same model, the regression coefficient for MNA more or equal to 24 compared to MNA less than 24 was −3.1 (95% CI: −4.8 to −1.4, *p* < 0.001) for chair stand test time (seconds). The regression coefficient for males compared to females was −1.8 (95% CI: −3.2 to −0.3, *p* = 0.019) for chair stand test time (seconds) (Table 4).

## 4. Discussion

This study found a strong link between specific dietary components and overall nutritional adequacy and muscle strength and functional performance, underscoring the central role of nutrition, especially sea vegetables and fruit, can play in the prevention and management of sarcopenia. It also found an association between care setting and these dependent variables.

Sarcopenia, whose risk factors include inactivity, malnutrition, smoking, extreme sleep duration, and diabetes, has been associated with poorer survival, postoperative complications, prolonged hospitalization, falls, fractures, metabolic disorders, cognitive decline, and increased mortality [1]. One systematic review and meta-analysis of community-dwelling older adults reported significant associations between sarcopenia and age (OR = 1.12, 95% CI: 1.10–1.13), underweightedness (OR = 3.78, 95% CI: 2.55–5.60), and malnutrition or risk of malnutrition (OR = 2.99, 95% CI: 2.40–3.72) as well as a range of disease-related conditions, including diabetes, cognitive impairment, osteoporosis, osteoarthritis, depression, falls, anorexia, and anemia [14]. A recent large-scale systematic review and meta-analysis applying the Asian Working Group for Sarcopenia (AWGS) 2019 diagnostic criteria reported the prevalence of sarcopenia among community-dwelling older adults (≥60 years) in Asia to be 16.5% (95% CI: 14.7–18.4%)**,** suggesting a substantial public health burden in the region [15].

Sarcopenia is increasingly viewed as a functional state shaped by multiple influences across aging. Physical activity is commonly recognized as an important determinant of muscle mass, strength, and physical performance. Lower habitual physical activity has been associated with accelerated functional decline in older adults, whereas exercise interventions—particularly resistance training—has been found able to improve key outcomes related to sarcopenia [16,17]. Moreover, exercise interventions have been shown to significantly improve muscle strength and physical performance in older adults already diagnosed with sarcopenia [18]. Diet and nutrition as well as setting have less often been directly tied to sarcopenia.

This cross-sectional study investigated the association between dietary and nutritional factors and sarcopenia among older adults in southern Taiwan. For sarcopenia, the adjusted odds ratio (OR) was 0.48 (95% CI 0.28–0.81) for those with a high school education level or above compared to those with lower education levels. More importantly, we found an independent association between daily intake of sea vegetables and fresh fruits and lower risk of sarcopenia (adjusted OR 0.38, 95% CI: 0.20–0.74) and 0.28, 95% CI: 0.16–0.49).

Sea vegetables are nutritionally dense foods providing protein (often 11–32% of dry weight), essential amino acids, minerals, vitamins, soluble fiber, and distinct marine bioactive. Their key compounds include fucoidan (a sulfated polysaccharide), phlorotannin (marine polyphenols), and fucoxanthin (a xanthophyll carotenoid). Collectively, these constituents exhibit anti-inflammatory, antioxidant, metabolic, and in some models anabolic/myogenic activities mechanistically relevant to sarcopenia. Recent reviews of edible seaweed nutrition and human trials report their benefits on glycemia, blood pressure, body composition and select functional markers, supporting the biological plausibility that routine seaweed intake can contribute to healthier aging trajectories when integrated into balanced diets [8]. Notably, a recent clinical and preclinical trial found that intake of Ishige okamurae, a brown seaweed, could help improve muscle strength and regeneration in both older adults and aging mice, with no evidence of toxicity, supporting the role of consuming edible seaweeds as a nutritional strategy for protecting against age-related muscle loss [6]. Recent reviews report the benefits of seaweed consumption on glycemic control, blood pressure, body composition, and functional outcomes [19]. The present study adds to this growing body of literature suggesting that regular integration of sea vegetables into the diet may help promote healthier aging.

Our findings indicate that sea vegetable intake exerts protective effects against sarcopenia, as daily consumption was significantly associated with a reduced risk of sarcopenia (adjusted OR = 0.38, *p* = 0.004) and greater grip strength (β = 1.9, *p* = 0.002). Supporting evidence from experimental studies shows that seaweed-derived compounds such as *Pyropia yezoensis* protein (PYCP) and fucosterol can attenuate TNF-α-induced or age-related muscle atrophy by downregulating inflammatory and proteolytic pathways (NF-κB, atrogin-1, MuRF1) and activating PI3K/Akt/mTOR signaling [5,7,20]. Although these mechanisms were demonstrated in cell and animal models only, they provide plausible biological support for our population-based findings. Moreover, macroalgae contain diverse bioactive compounds, including polyphenols, carotenoids, and fucoidans [21]. Further clinical studies are needed to clarify their roles in preventing sarcopenia.

This study also identified a significant association between daily fruit consumption and reduced sarcopenia risk, suggesting that antioxidants may contribute to muscle preservation. Consistent with our findings, a meta-analysis of 14 observational studies reported an inverse association between combined fruit and vegetable intake and sarcopenia (OR = 0.61, 95% CI: 0.48–0.79) [22], and large-scale studies have shown similar protective effects of fruit intake, particularly among women [1,23]. While most evidence to date has been observational, these findings collectively support the potential role of fruit-derived antioxidants in maintaining muscle health. In vitro studies further provide biological plausibility, showing that nutrients and phytochemicals such as vitamin C, phloretin, carotenoids, quercetin, and kaempferol can reduce oxidative stress and promote myogenic activity in muscle cells [9,10,24,25,26].

**Figure 1:**
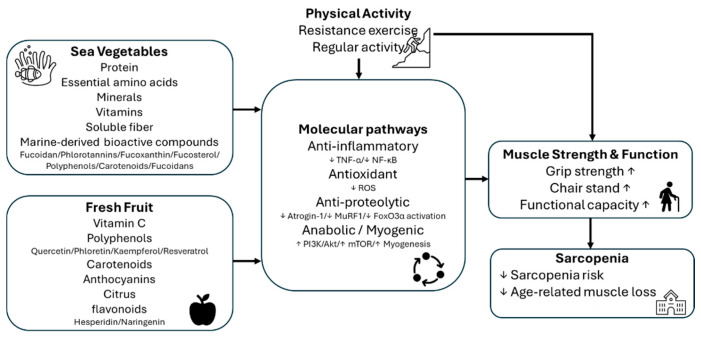
Proposed dietary and mechanistic pathways linking sea vegetables and fresh fruits to reduce sarcopenia risk. Arrows represent increases (upward) or decreases (downward) in the corresponding variables shown.

Sea vegetables and fresh fruits are broad categories encompassing diverse phytochemical profiles, making it important to differentiate by type for nutritional interpretation. Different sea vegetable groups (e.g., brown, red, and green algae) vary in their expression of bioactive compounds. For example, fucosterol and fucoxanthin in brown algae modulate the PI3K/Akt/mTOR and FoxO3α pathways in muscle cells [5,7,21], while sea-vegetable polyphenols and carotenoids contribute additional antioxidant and anti-inflammatory activity. Similarly, fresh fruits differ markedly in their dominant phytochemicals. For example, grapes contain quercetin and resveratrol, berries are rich in anthocyanins, citrus fruits provide hesperidin and naringenin, and apples include phloridzin and quercetin. These differences likely influence key processes in muscle metabolism, such as oxidative stress suppression, inflammatory cytokine attenuation, protein synthesis activation (e.g., mTOR signaling), and protein-degradation prevention (e.g., MuRF1/atrogin-1 inhibition) [9,10,24,25,26]. Thus, distinguishing food subtypes improves mechanistic insight, better explains variability in epidemiological results, and enables more precise dietary recommendations and intervention design. A schematic diagram summarizing the proposed dietary–mechanistic pathways linking sea vegetables and fruits to sarcopenia prevention is presented in Figure 1. However, these mechanistic explanations were derived from cell and animal models, so they should be interpreted as supportive biological evidence rather than direct confirmation in humans. Further intervention studies are needed to validate these potential pathways.

In this study, nutritional adequacy emerged as a significant predictor of functional performance, even though MNA scores did not differ significantly between participants with and without sarcopenia. Specifically, better MNA status was associated with higher grip strength (per kg) and faster chair stand performance, indicating that nutritional status may influence muscle quality and physical function before overt declines in muscle mass become apparent. This relationship aligns with previous findings showing that suboptimal nutrition contributes to functional impairment and mobility limitation in older adults [27,28,29]. Rather than serving solely as a diagnostic correlation of sarcopenia, the MNA appears to function as an indicator of functional reserve and physiological resilience. These observations underscore the importance of incorporating nutritional screening into sarcopenia prevention strategies. It can be used to identify those at high risk and intervene in sarcopenia development before functional decline progresses.

Participants in this study were primarily recruited from hospital outpatient clinics and a home care center, which may not fully represent community-dwelling older adults. The inclusion of both outpatient and institutionalized populations could introduce heterogeneity in health status, comorbidity burden, and lifestyle factors. Such heterogeneity may partly explain the differences in sarcopenia prevalence we observed across study sites. For instance, patients in outpatient clinics might represent healthier and more mobile older adults, whereas those enrolled from a home care center may present with greater functional impairments or higher chronic disease burden. These differences could influence dietary behaviors, physical activity levels, and access to nutritional support, thereby contributing to variability in sarcopenia outcomes between institutions. Older adults recruited from the veteran-affiliated tertiary hospital and its home-care center were likely to have had greater multimorbidity, lower mobility, and poorer nutritional status than outpatient attendees at the regional hospital. These differences may partly account for the higher sarcopenia risk in patients enrolled from those sites.

### Study Limitations

This study has some limitations. Although we tried to minimize selection bias by recruiting participants from institutions of varying sizes and across different geographic regions, one limitation of this study is potential selection bias due to its use of a cross-sectional data to compare individuals with and without sarcopenia. Because participants were recruited from hospital outpatient departments and a home-care center, our sample represents a clinical cohort and may not reflect the general community-dwelling older population. This limitation should be considered when interpreting the generalizability of the findings. Another possible limitation is related to the biochemical data we collected. Our biochemical data were obtained from different hospital units and laboratories. Although all institutions follow standardized quality control procedures, there may have been some minor inter-laboratory variation. Another limitation is potential recall bias. To reduce this possibility, we required that the questionnaires be administered by trained interviewers using standardized protocols in a controlled, distraction-free environment. Standardized questionnaires and relevant blood tests were utilized to further reduce the impact of recall bias. Finally, because this study had a cross-sectional design, the associations observed cannot establish causality, and longitudinal or interventional studies are needed to confirm these findings.

## 5. Conclusions

In summary, we found intake of sea vegetables and fruits, which contain bioactive compounds and antioxidants that may modulate inflammation, oxidative stress, and anabolic pathways relevant to muscle preservation, along with nutritional adequacy and institutional differences to be significant determinants of sarcopenia and physical function among older adults in Taiwan. These results extend prior research by identifying specific food groups with potential protective effects in Asian clinical settings and they underscore the importance of promoting nutrition-focused interventions, ones encouraging the consumption of sea vegetables and fruits, ensuring nutritional adequacy, as strategies to reduce sarcopenia risk in aging populations, especially in Asian populations in areas where these foods are readily available. Further longitudinal studies are warranted to confirm these associations.

## Figures and Tables

**Table 1:** Macronutrient composition of selected fresh sea vegetables and tropical fruits. (per 100 g edible portion).

Food Category	Food Item	Energy (kcal)	Protein (g)	Fat (g)	Carbohydrates (g)	Dietary Fiber (g)
Seaweed	Kelp (fresh)	14	0.7	0.1	4.2	2.5
Seaweed	Wakame (fresh)	39	5.0	0.0	11.6	10.1
Seaweed	Kelp strip (fresh)	17	0.7	0.1	5.0	2.8
Seaweed	Black moss (dry)	275	10.3	0.5	69.5	14.6
Seaweed	Laver, nori (dry)	212	28.1	0.9	47.9	29
Fruit	Banana	82	1.5	0.1	22.1	1.6
Fruit	Grape	68	0.4	0.4	17.7	0.5
Fruit	Lychee	64	1.0	0.2	16.5	0.8
Fruit	Longan	69	1.1	0.4	18.0	1.8
Fruit	Mango	48	0.9	0.2	13.0	1.2

All nutrient values were obtained from the Taiwan Food and Drug Administration (TFDA). Food Nutrition Database (latest version, accessed 2025). Only fresh (wet-weight) edible forms were included; dried, powdered, or processed products were excluded.

**Table 2:** The characteristics of subjects with or without sarcopenia.

	With Sarcopenia	Without Sarcopenia	
N	159	429	*p*-Value
Age (years old)	81.1 ± 14.6	81.8 ± 17.8	0.20
Gender, n (%)			0.12
Male	78 (49.1)	206 (48.0)	
Female	81 (50.9)	223 (52.0)	
Smoking, n (%)			<0.001
Never	144 (90.6)	334 (77.9)	
Former	7 (4.4)	72 (16.8)	
Current	8 (5.0)	23 (5.4)	
Alcohol, n (%)			0.002 ^a^
Never	156 (98.1)	385 (89.7)	
>1 per week	3 (1.9)	36 (8.4)	
>2 per week	0 (0.0)	8 (1.9)	
Betel chewing, n (%)			0.019 ^a^
Never	157 (98.7)	403 (93.9)	
Former	1 (0.6)	22 (5.1)	
Current	1 (0.6)	4 (0.9)	
Education level, n (%)			
Higher school and more	42 (26.4)	178 (41.5)	<0.001
BIA (%)	8.6 ± 10.1	9.3 ± 1.3	0.2
Grip (Kg)	17.9 ± 7.0	20.4 ± 7.6	<0.001
Six-meter gait speed (meter/second)	0.6 ± 0.3	0.9 ± 0.7	0.001
Chair stand test time (second)	15.8 ± 11.7	12.6 ± 11.7	0.004
Fish paste products (fish balls, fish soup), n (%)			
Three per month	37 (23.3)	73 (17.0)	0.08
Canned fish, n (%)			
Used	68 (42.8)	136 (31.7)	0.012
Dried small fish with bones (Kissed larvae, dried small fish), n (%)			
Three per month	65 (40.9)	103 (24.0)	<0.001
Other non-fish seafood (shrimp, flower sticks, hairy crab), n (%)			
Three per month	17 (10.7)	82 (19.1)	0.015
Livestock and poultry offal (chicken liver, kidney), n (%)			
Used	32 (20.1)	128 (29.8)	0.019
Processed meat products (gong balls, hot dogs), n (%)			
Used	74 (46.5)	186 (43.4)	0.49
Light-colored vegetables (radish, Chinese cabbage), n (%)			0.001
Four to six per week	18 (11.3)	69 (16.1)	
per day	44 (27.7)	174 (40.6)	
Sea vegetables (algae) (sea vegetables, seaweed, hair vegetables), n (%)			
Used	27 (17.0)	185 (43.1)	<0.001
Canned and salted frozen vegetables, n (%)			
Used	13 (8.2)	88 (20.5)	<0.001
Fresh fruits (grapes, bananas, lychees, longan), n (%)			
Used	57 (35.8)	293 (68.3)	<0.001
Green tea, n (%)			
Used	5 (3.1)	81 (18.9)	<0.001
Black coffee, n (%)			
per day	18 (11.3)	56 (13.1)	0.57
Mini Nutritional Assessment (MNA), n (%)			
≥24	111 (69.8)	317 (73.9)	0.32
Biochemical			
Albumin (mg/dL)	4.7 ± 3.7	5.1 ± 4.1	0.35
Blood Urea Nitrogen (mg/dL)	20.1 ± 10.6	22.3 ± 13.0	0.06
Serum creatinine (mg/dL)	2 ± 2.5	1.9 ± 3.0	0.88
Glomerular filtration rate (min/mL/1.73 m^2^)	65.5 ± 35.0	61.6 ± 30.7	0.20
Uric acid (mg/dL)	5.7 ± 1.9	6 ± 1.9	0.039
Ante Cibum sugar (mg/dL)	135.1 ± 79.1	152.6 ± 99.0	0.05
Hemoglobin A1c (%)	7.1 ± 1.4	7.2 ± 1.4	0.47
Total cholesterol (mg/dL)	174.9 ± 37.4	167.9 ± 36.4	0.044
Triglyceride (mg/dL)	115.1 ± 70.3	119.4 ± 64.9	0.49
Systolic blood pressure (mmHg)	139 ± 22	138 ± 22	0.75
Diastolic blood pressure (mmHg)	73 ± 10	73 ± 15	0.99
Enroll institution			<0.001
Kaohsiung Veterans General Hospital, n (%)	36 (22.6)	221 (51.5)	
Pingtung Veterans General Hospital Longquan Branch, n (%)	12 (7.5)	89 (20.7)	
Pingtung Hospital, n (%)	111 (69.8)	119 (27.7)	

^a^: Fisher’s exact test.

**Table 3:** The factors associated with sarcopenia risk in the multivariate logistic regression model.

	Crude Model		Full Model ^a^	
	Crude OR (95% Confidence Interval)	*p*-Value	Adjusted OR (95% Confidence Interval)	*p*-Value
Age (per years old)	1.00 (0.99–1.01)	0.68	1.01 (1.00–1.03)	0.11
Male vs. female	1.04 (0.72–1.50)	0.82	1.63 (0.98–2.70)	0.06
Education level				
Higher school and more	0.51 (0.34–0.76)	0.001	0.48 (0.28–0.81)	0.006
Sea vegetables (algae) (sea vegetables, seaweed, hair vegetables)				
Used vs. non-used	0.27 (0.17–0.43)	<0.001	0.38 (0.20–0.74)	0.004
Light-colored vegetables (radish, Chinese cabbage)				
Less than 4 per week	1.0 (reference)		1.0 (reference)	
Four to six per week	0.50 (0.28–0.89)	0.018	1.01 (0.48–2.11)	0.99
per day	0.48 (0.32–0.73)	0.001	1.30 (0.70–2.42)	0.41
Fresh fruits (grapes, bananas, lychees, longan)				
Less than one serving per day	1.0 (reference)		1.0 (reference)	
per day	0.26 (0.18–0.38)	<0.001	0.28 (0.16–0.49)	<0.001
Black coffee				
Less than three cups per week	1.0 (reference)		1.0 (reference)	
Four to six or more per week	0.85 (0.48–1.50)	0.57	1.19 (0.58–2.44)	0.63
Mini Nutritional Assessment (MNA)				
≥24 vs. <24	0.82 (0.55–1.22)	0.32	0.70 (0.40–1.20)	0.19
Glomerular filtration rate (per 10 min/mL/1.73 m^2^)	1.08 (1.01–1.15)	0.029	1.11 (1.02–1.20)	0.011

^a^: adjusted age, gender, education level, dried small fish with bones (kissed larvae, dried small fish), fish paste products (fish balls, fish soup), canned fish, other non-fish seafood (shrimp, flower sticks, hairy crab), processed meat products (gong balls, hot dogs), livestock and poultry offal (chicken liver, kidney), light-colored vegetables (radish, Chinese cabbage), sea vegetables (algae) (sea vegetables, seaweed, hair vegetables), canned and salted frozen vegetables, fresh fruits (grapes, bananas, lychees, longan), green tea, Mini Nutritional Assessment (MNA) and glomerular filtration rate in multiple logistic regression model.

**Table 4:** The factors associated with grip (per kg), six-meter walk time, and up and go time of the subjects in the multiple linear regression model.

	Grip (per kg)		6 m Gait Speed (per Meter/Second)		Chair Stand Test Time (per Second)	
	Regression Coefficients (95% Confidence Intervals) ^a^	*p* Value	Regression Coefficients (95% Confidence Intervals) ^a^	*p* Value	Regression Coefficients (95% Confidence Intervals) ^a^	*p* Value
Age (per years old)	<0.1 (<0.1 to 0.0)	0.007	<0.1 (<0.0 to 0.1)	0.30	<0.1 (<0.1 to 0.1)	0.12
Male vs. female	8.1 (7.1 to 9.2)	<0.001	−0.1 (−0.2 to 0.1)	0.41	−1.8 (−3.2 to −0.3)	0.019
Education level						
Higher school and more	2.0 (0.9 to 3.1)	<0.001	0.1 (−0.1 to 0.2)	0.21	−0.8 (−2.4 to 0.7)	0.28
Sea vegetables (algae) (sea vegetables, seaweed, hair vegetables)						
Used vs. non-used	1.9 (0.7 to 3.1)	0.002	0.2 (0.1 to 0.4)	0.003	1.1 (−0.6 to 2.7)	0.21
Light-colored vegetables (radish, Chinese cabbage)						
Four-six servings week	−1.3 (−2.9 to 0.4)	0.13	0.2 (0.0 to 0.4)	0.039	−0.9 (−2.5 to 0.6)	0.25
per day	−0.5 (−1.7 to 0.8)	0.46	−0.1 (−0.3 to 0.1)	0.22	0.1 (−1.6 to 1.9)	0.89
Fresh fruits (grapes, bananas, lychees, longan)						
per day	0.8 (−0.6 to 2.2)	0.25	−0.2 (−1.3 to 1.0)	0.79	1.7 (−0.1 to 3.4)	0.06
Green tea						
4–6 cups or more per week	0.3 (−1.2 to 1.8)	0.69	0.2 (−1.1 to 1.6)	0.71	1.0 (−1.1 to 3.0)	0.35
Black coffee						
4–6 cups or more per week	1.3 (−0.1 to 2.8)	0.07	−0.2 (−1.5 to 1.1)	0.75	−1.0 (−3.0 to 1.0)	0.31
Mini Nutritional Assessment (MNA)	3.1 (1.8 to 4.3)	<0.001	−0.3 (−0.5 to −0.1)	0.12	−6.1 (−8.6 to −3.5)	<0.001
≥ 24 vs. < 24	2.9 (1.6 to 4.1)	<0.001	0.3 (−0.8 to 1.4)	0.61	−3.1 (−4.8 to −1.4)	<0.001
Glomerular filtration rate (per 10 min/mL/1.73 m^2^)	0.3 (0.1 to 0.4)	0.003	<0.1 (−0.1 to 0.2)	0.65	−0.9 (−1.1 to −0.6)	<0.001

^a^: model adjusted for age, gender, education level, dried small fish with bones (kissed larvae, dried small fish), fish paste products (fish balls, fish soup), canned fish, other non-fish seafood (shrimp, flower sticks, hairy crab), processed meat products (gong balls, hot dogs), livestock and poultry offal (chicken liver, kidney), light-colored vegetables (radish, Chinese cabbage), sea vegetables (algae) (sea vegetables, seaweed, hair vegetables), canned and salted frozen vegetables, fresh fruits (grapes, bananas, lychees, longan), green tea, Mini Nutritional Assessment (MNA) and glomerular filtration rate in multivariable linear regression model.

## Data Availability

The data presented in this study are available on request from the corresponding author due to restrictions related to participant confidentiality.

## Abbreviations

The following abbreviations are used in this manuscript:

AWGS	Asian Working Group for Sarcopenia
BIA	bioelectrical impedance analysis
BMI	body mass index
CI	confidence interval
eGFR	estimated glomerular filtration rate
KVGH	Kaohsiung Veterans General Hospital
MNA	Mini Nutritional Assessment
OR	odds ratio
sd	standard deviation
SMI	skeletal muscle mass index
TSMH	Antai Tian-Sheng Memorial Hospital
